# Peruvians’ sleep duration: analysis of a population-based survey on adolescents and adults

**DOI:** 10.7717/peerj.345

**Published:** 2014-04-10

**Authors:** Rodrigo M. Carrillo-Larco, Antonio Bernabé-Ortiz, J. Jaime Miranda, Jorge Rey de Castro

**Affiliations:** 1 CRONICAS Center of Excellence in Chronic Diseases, Universidad Peruana Cayetano Heredia, Lima, Peru; 2 Department of Medicine, School of Medicine, Universidad Peruana Cayetano Heredia, Lima, Peru; 3 Grupo de Investigación en Sueño (GIS), Lima, Peru

**Keywords:** Sleep, Peru, Cross-sectional studies, Sleep deprivation, Sleep duration, Time-use studies, Socioeconomic factors

## Abstract

**Background**. Sleep duration, either short or long, has been associated with diseases such as obesity, type-2 diabetes and cardiovascular diseases. Characterizing the prevalence and patterns of sleep duration at the population-level, especially in resource-constrained settings, will provide informative evidence on a potentially modifiable risk factor. The aim of this study was to explore the patterns of sleep duration in the Peruvian adult and adolescent population, together with its socio-demographic profile.

**Material and Methods**. A total of 12,424 subjects, mean age 35.8 years (SD ±17.7), 50.6% males, were included in the analysis. This is a cross-sectional study, secondary analysis of the Use of Time National Survey conducted in 2010. We used weighted means and proportions to describe sleep duration according to socio-demographic variables (area and region; sex; age; education attainment; asset index; martial and job status). We used Poisson regressions, taking into account the multistage sampling design of the survey, to calculate crude and adjusted prevalence ratios (PR) and 95% confidence intervals (95% CI). Main outcomes were short- (<6 h) and long-sleep duration (≥ 9 h).

**Results**. On average, Peruvians slept 7.7 h (95% CI [7.4–8.0]) on weekdays and 8.0 h (95% CI [7.8–8.1]) during weekends. The proportions of short- and long-sleep, during weekdays, were 4.3% (95% CI [2.9%–6.3%]) and 22.4% (95% CI [14.9%–32.1%]), respectively. Regarding urban and rural areas, a much higher proportion of short-sleep was observed in the former (92.0% vs. 8.0%); both for weekdays and weekends. On the multivariable analysis, compared to regular-sleepers (≥ 6 to <9 h), short-sleepers were twice more likely to be older and to have higher educational status, and 50% more likely to be currently employed. Similarly, relative to regular-sleep, long-sleepers were more likely to have a lower socioeconomic status as per educational attainment.

**Conclusions**. In this nationally representative sample, the sociodemographic profile of short-sleep contrasts the long-sleep. These scenarios in Peru, as depicted by sleeping duration, differ from patterns reported in other high-income settings and could serve as the basis to inform and to improve sleep habits in the population. Moreover, it seems important to address the higher frequency of short-sleep duration found in urban versus rural settings.

## Introduction

Over the last decades, sleep duration has changed: people are sleeping less (or more) than they used to. A recent study analyzed data from the 1970s to the 2000s of ten industrialized countries, i.e., Australia, Canada, Finland, Germany, Italy, Netherlands, Norway, Sweden, United Kingdom, United States, and concluded that long-sleep duration, over nine hours, was more prevalent (Bin, Marshall & Glozier, 2013). However, a systematic review of cross-sectional studies conducted between the 1960s and 2000s in 15 countries reported a mixed trend: whilst seven countries, i.e., Bulgaria, Poland, Canada, France, Britain, Korea and the Netherlands had an increased sleep duration, six, i.e., Japan, Russia, Finland, Germany, Belgium and Australia had a reduced one (Bin, Marshall & Glozier, 2012).

Inadequate sleep duration, either in excess or deficit, has been associated with cardiovascular diseases and other non-communicable diseases (NCDs). A meta-analysis of prospective studies reported that either short- or long-sleep duration: (1) is a risk factor for dying of coronary heart disease or stroke (Cappuccio et al., 2011); (2) is associated with hypertension (Guo et al., 2013); (3) is associated with type-2 diabetes (Cappuccio et al., 2010a); (4) is associated with obesity (Marshall, Glozier & Grunstein, 2008). In general, individuals with short- or long-sleep patterns are at higher risk of all cause mortality (Cappuccio et al., 2010b; Gallicchio & Kalesan, 2009); yet, the evidence is not conclusive on this matter (Kurina et al., 2013).

Determining how much time a person sleeps is important for elucidating newer avenues for prevention as it could provide a practical target of a risk factor amenable to modification. Unfortunately there is limited data published on this matter in Latin American countries. Recent systematic reviews or meta-analysis on sleep patterns, sleep duration and its association with socio-demographic variables have not included any Latin American countries (Bin, Marshall & Glozier, 2013; Galland et al., 2012), with the exception of one effort that included Brazil with a study that targeted adolescents (Olds et al., 2010).

There are important reasons to determine sleep duration profiles in the Peruvian population as well as in other emerging countries given their context-specific environments of rapid transitioning societies with important dual burdens of infectious diseases and chronic conditions. These context-specific settings will certainly have a direct impact on the profile of risk factors for short- or long-sleep duration as well as on the profile of conditions that are associated with sleep restriction. First, Peru is undergoing an epidemiological transition with an increasing prevalence of NCDs (Huicho et al., 2009), and this phenomena, paired with economic development, will certainly impact the profile of sleeping patterns as well as its relationship with other diseases. Second, another context-specific characteristic from our sample is the road traffic injuries that are linked to tiredness or sleepiness (Rey de Castro & Rosales-Mayor, 2010), which is a very common feature among Lima’s public transportation drivers (Risco et al., 2013). Thus, having a broader picture at the population-level of sleeping-related factors would clarify the potential scope of this public health problem. Third, and linked to the previous argument, home injuries could be preventable events, especially among the elderly population, as some reports suggest that people aged 75 years and over and who were long-sleepers were more likely to suffer falls (Mesas, Lopez-Garcia & Rodriguez-Artalejo, 2011). Finally, from a different and yet related angle, maternal and child health remain an area of public health priority in the developing world and sleep duration may play a role as it may be associated with pre-term delivery and post-partum depression (Chang et al., 2010). These are some examples of how approaching sleeping patterns in low- and middle-income settings, where information at a general population level is lacking, could well inform and close existing knowledge gaps.

The aim of this study was to characterize the patterns of sleep duration in the Peruvian population, and to describe the socio-demographic profile of short- and long-sleepers using a nationally representative cross-sectional survey.

## Methods

### Study design and participants

This is a secondary analysis of a population-based survey. The data came from the Use of Time National Survey (*Encuesta Nacional de Uso del Tiempo-ENUT* in Spanish) conducted by the Peruvian National Institute of Statistics and Informatics (INEI) in the year 2010. Information about this survey is publicly available online (Instituto Nacional de Estadística e Informática, 2010a; Instituto Nacional de Estadística e Informática, 2010b; Instituto Nacional de Estadística e Informática, 2010c).

The original survey had a random sample of participants, drawn using standard probabilistic two-step procedures: clusters (primary sampling units) and households (secondary sampling units). The final sample included 4580 households grouped up in 510 clusters: 3080 houses were from urban and 1500 from rural areas.

The study population consisted of all permanent residents and those living in the selected household at the moment of the survey. They recorded information on personal needs, including sleep duration of participant’s aged 12 or above. People living in institutionalized collective residences, i.e., hospitals or jails, were excluded.

### Questionnaire & proceedings

Details of the ENUT questionnaire, sections and contents are available elsewhere (Instituto Nacional de Estadística e Informática, 2010a; Instituto Nacional de Estadística e Informática, 2010b; Instituto Nacional de Estadística e Informática, 2010c). We included the following information in this secondary analysis: (a) household characteristics (type of house, main wall material, main floor material, total number or rooms, total number of bedrooms, water source, sewage management, and sharing bathroom), and assets (iron, blender, radio, television, washing machine, dryer machine, computer, telephone, Internet, car); (b) household members’ characteristics (sex, age, marital status, and educational attainment); (c) activities for the household that include personal needs (sleep duration); and (d) job status (employment status the week prior to the survey).

A trained fieldworker, who visited each of the selected households, administered the survey. The interviewer contacted the participant, then explained the aim of the study and ensured the confidentiality of the survey. In order to avoid comprehension bias, fieldworkers read the questions as they were written. They asked all the participants about the activities they performed in a 24-h period taking as a reference the week before the interview; that is, the last Monday–Friday and Saturday–Sunday period. The survey was conducted between November 15th and December 30th, 2010 (Instituto Nacional de Estadística e Informática, 2010a; Instituto Nacional de Estadística e Informática, 2010b; Instituto Nacional de Estadística e Informática, 2010c). Vacations in Peru, in particular school vacations, are from January to March; the timing of the survey did not overlap with our vacations.

### Variables

The outcome variable for this study was the total number of hours the participant self-reported or slept during the week before the survey, assessed through the question: “*how many hours did you sleep from Monday to Friday?”* For analysis purposes, and assuming that participants had similar sleeping hours every day, to calculate the average number of hours the participant slept daily we divided the total number of hours the participant slept during the previous week by five. For weekends, we proceeded in the same way, dividing total hours by two. Afterwards, these variables were categorized as follows: short-sleep (<6 h per day), regular-sleep (from 6 to less than 9 h per day), and long-sleep (9 and more hours). The rationale for choosing these cut-off points was based on a recent critical review on sleep duration and all-cause mortality that included a study that reported an elevation in mortality risk, for men and women and in a U-shaped curve, using the chosen sleep categories (Kurina et al., 2013). Furthermore, according to the National Sleep Foundation an adult needs 7–9 h of sleep, while teens (10–17 years old) need 8.5–9.25 h (National Sleep Foundation). We used the same sleep duration definition for all ages. We took this decision because sleeping less than six hours is considered to be short sleep duration for both teens and adults, as per recommendation of The National Sleep Foundation. We understand that teens (aged 10 to 17) should sleep between 8.50–9.25 h, so our definition is short by 15 min. However, we consider this a minor issue because only 436 observations that belong to teens sleep over 9.15 h.

The ENUT survey inquired about several types of daily activities, with the premise that all of them should add up to 24 h. We conducted verification analysis of these sum procedures using the whole dataset and the main results presented in the ENUT’s final report (Instituto Nacional de Estadística e Informática, 2010a; Instituto Nacional de Estadística e Informática, 2010b; Instituto Nacional de Estadística e Informática, 2010c). After adding all the activities presented in the final report, the result was on average 31.95 h (per day); though this number is most likely to be overestimated, because activities that are not performed in a daily basis nor every week (e.g., buying new clothes/shoes, or buying spare parts for home appliances) were included. Furthermore, after considering only activities that are more likely to be done during a regular day or week (e.g., sleep, eat, or work) the result was 23.90 h per day. In so doing, we believe the estimates on sleep duration are accurate enough for the purposes of this study.

Additional variables considered for the socio-demographic characteristics of the participants were area (rural or urban); region (Lima, rest of the Coast, Highlands, and the Amazon Region); gender (male, female); age (12–19, 20–35, 36–64, ≥ 65 years); education (none/primary school, high school, higher); asset index (in tertiles); job status (yes or no depending upon the participant had worked the week previous to the survey), and marital status (single, cohabiting partner/married, separated/widow/divorced).

We constructed the indicator asset index from the module of the survey comprising household characteristics and assets, according to Gordon’s proposed methodology (Gordon & Pantazis, 1997). The variables included in the index (Cronbach’s alpha >0.80) were: type of house; main wall material; main floor material; total number or rooms; total number of bedrooms; household water source; sewage service at bathroom; if the bathroom was shared or not; and assets (iron, blender, radio, television, washing machine, dryer machine, computer, telephone, Internet, car).

### Statistical methods

We conducted the analysis with STATA 11.0 (StataCorp, College Station, TX, USA). For all calculations and estimations (results and all three tables) reported, we used the *SVY* command provided the multistage design, based upon area and region variables of the ENUT. We used appropriate techniques for estimating results in subpopulations of interest, to guarantee accurate calculation of standard errors and, hence, inference of our findings we used the *SUBPOP* command in the Poisson regression models (West, Berglund & Heeringa, 2008). We calculated means, standard deviations and percentages for continuous and categorical variables, respectively. We conducted T-test and Chi-squared test to assess differences between continues and categorical variables. To assess associations with the outcomes of interest, we used Poisson regression and report prevalence ratios (PR) and 95% confidence intervals (95% CI). For the multivariable model we utilized a stepwise backward technique (we included all variables in a model, those with a *p*-value >0.05 for the Wald Test were dropped out of the model) and report variables independently associated with the outcomes of interest. Throughout the analysis we considered a *p* < 0.05 to be statistical significant (Bonferroni correction for 12 comparisons: 0.004).

### Ethics

This is a secondary-data analysis of a publicly-available dataset stored at a public national repository (Instituto Nacional de Estadística e Informática, 2010a; Instituto Nacional de Estadística e Informática, 2010b; Instituto Nacional de Estadística e Informática, 2010c); so, approval from an Institutional Review Board was not considered mandatory. The dataset used does not provide any kind of information that might have allowed us, or any other researcher, to identify participants of the study, ensuring confidentiality.

## Results

### Sample characteristics

There were 18,412 observations in the original dataset and 5,988 (32.5%) were excluded due to missing values in the outcome of interest; thus, the final sample was 12,424. A detailed comparison of those participants with missing data and those included in the analysis is shown in Table 1 (complete data was found for marital status); a significant difference was found with the variables region and sex. The mean age was 35.8 years (SD: ±17.7) and there were almost a similar proportion of men and women. Details on the sample characteristics are also shown in Table 1.

**Table 1:** Participants’ characteristics (*n* = 12,424).

Variable	%
**Area**	
Urban	75.8
Rural	24.2
**Region**	
Highlands	31.7
Coast (except Lima)	24.0
Amazon	12.1
Lima	32.2
**Sex**	
Male	50.1
Female	49.9
**Age**	
12–19	20.6
20–35	34.1
36–64	37.2
≥ 65	8.1
**Education**	
None/Primary	30.9
High school	45.8
Higher	23.3
**Assets index**	
Lowest	23.8
Middle	34.4
Highest	41.9

### Sleep duration

Peruvians reported to sleep 7.7 h (95% CI [7.4–8.0]) on average during weekdays and 8.0 h (95% CI [7.8–8.1]) during weekends (t-test between sleep duration during weekdays and weekends, *p* < 0.001). After categorizing this variable, during weekdays 4.3% (95% CI [2.9%–6.3%]), 73.4% (95% CI [65.8%–79.8%]), and 22.4% (95% CI [14.9%–32.1%]) would qualify as short-, regular-, and long-sleep respectively. For weekend periods these proportions were 4.1% (95% CI [3.2%–5.4%]), 65.4% (95% CI [59.4%–70.9%]), and 30.5% (95% CI [24.3%–37.5%]) for short-, regular, and long-sleep, respectively. Given the similar sleep duration in weekdays and weekends further analyses were conducted only with the weekdays’ data. Table 2 shows socio-demographic variables according to sleep duration categories.

**Table 2:** Distribution of self-reported sleep duration on weekdays by socio-demographic variables. ENUT Peru 2010.

Variable	Sleep duration (%)			*p*
	**Short-sleep**	**Regular sleep**	**Long-sleep**	
	*n* = 470	*n* = 8,877	*n* = 3,077	
**Area**				
Urban	5.2	76.9	17.9	0.003
Rural	1.4	62.4	36.2	
**Region**				
Highlands	2.8	67.9	29.3	0.031
Coast (except Lima)	5.1	74.5	20.4	
Amazon	2.2	66.0	31.8	
Lima	5.9	80.7	13.5	
**Sex**				
Male	4.5	73.5	22.0	0.460
Female	4.0	73.3	22.7	
**Age**				
12–19	1.5	58.5	40.1	<0.001
20–35	4.0	77.4	18.6	
36–64	6.1	80.1	13.8	
≥65	3.8	64.0	32.2	
**Education**				
None/Primary	3.0	63.9	33.0	<0.001
High school	3.6	75.0	21.4	
Higher	7.2	82.7	10.1	
**Assets index**				
Lowest	2.3	62.9	34.9	<0.001
Middle	4.0	72.2	23.8	
Highest	5.6	80.3	14.1	
**Marital status**				
Single	3.3	67.5	29.2	0.001
Married/Living together	4.4	77.5	18.1	
Separate/Divorced/Widowed	5.3	77.0	17.7	
**Job status**				
No	2.4	64.7	33.0	0.001
Yes	5.5	79.2	15.3	

### Sub-national analysis of sleep duration

Sleep duration was rather similar by location (urban and rural): mean sleep duration during weekdays and weekends, in urban areas was 7.6 and 7.9 h, respectively; in rural areas the values were 8.2 and 8.3 h, respectively. Overall, people living in urban settings sleep less. The t-test between sleep duration during weekdays and weekends, according to location, was statistically significant (*p* < 0.001).

The following results were obtained using the *subpop* command, first we considered males to be equal to zero and then this value was assigned to women; similarly was performed for the analysis with regard to location (rural or urban).

The proportion of short-sleepers among men was 4.5% (95% CI [3.4%–6.0%]) and with regard to women it was 4.0% (95% CI [2.3%–6.8%]). However, a different trend was seen for long-sleep: 22.7% (95% CI [15.5%-32.0%]) and 22.0 (95% CI [14.3%–32.3%]) for women and men, respectively.

Regarding urban and rural areas, a much higher proportion of short-sleep was observed in the former: 5.2% (95% CI [3.8%–7.0%]) for urban and 1.4 (95% CI [1.0%–2.1%]) for rural areas. However, the proportion of long-sleep was almost the double in rural versus urban areas: 36.2% (95% CI [30.1%–42.9%]) for the former and 17.9% (95% CI [11.3%–27.6%]) for the latter.

### Sleep duration profile

The socio-demographic profile of short- and long-sleep is presented in Table 3. All point estimates were attenuated, became closer to 1, in all calculations following adjustment for co-variables as detailed in the multivariable analysis shown in Table 3. Variables independently associated with short-sleep in the multivariable model were: age, education, and work. The higher probability of being a short-sleeper was correlated with older age and with current employment status. Those with high school or no education had a lower probability compared to those with higher education. On the other hand, variables independently associated with long sleep were: sex, age, education, asset index, work, and marital status.

**Table 3:** Associations between socio-demographic variables and self-reported sleep duration. ENUT Peru 2010^*^.

Variables	Crude	Multivariable^**^	Crude	Multivariable^***^
	Short- vs. regular-sleep	Short- vs. regular-sleep	Long- vs. regular-sleep	Long- vs. regular-sleep
	PR (95% CI)	PR (95% IC)	PR (95% IC)	PR (95% IC)
**Sex**				
Male	1 (Reference)		1 (Reference)	1 (Reference)
Female	0.89 (0.62–1.28)		1.03 (0.93–1.13)	**0.88 (0.82**–**0.94)**
**Age**				
12–19	1 (Reference)	1 (Reference)	1 (Reference)	1 (Reference)
20–35	**2.03 (1.26**–**3.27)**	1.43 (0.98–2.08)	**0.48 (0.37**–**0.62)**	**0.79 (0.71**–**0.87)**
36–64	**2.92 (1.39**–**6.16)**	**2.18 (1.30**–**3.67)**	**0.36 (0.24**–**0.55)**	**0.60 (0.50**–**0.72)**
≥ 65	**2.32 (1.33**–**4.04)**	**2.24 (1.22**–**4.11)**	0.82 (0.56–1.21)	0.99 (0.77–1.28)
**Education**				
Higher	1 (Reference)	1 (Reference)	1 (Reference)	1 (Reference)
High school	**0.56 (0.48**–**0.66)**	**0.53 (0.32**–**0.86)**	**2.04 (1.88**–**2.22)**	**1.42 (1.34**–**1.51)**
None/Primary	**0.56 (0.39**–**0.80)**	**0.63 (0.57**–**0.70)**	**3.14 (2.27**–**4.34)**	**2.17 (1.72**–**2.73)**
**Assets index**				
Lowest	1 (Reference)		1 (Reference)	1 (Reference)
Middle	1.47 (0.79–2.75)		**0.69 (0.54**–**0.89)**	**0.77 (0.63**–**0.94)**
Highest	**1.84 (1.03**–**3.28)**		**0.42 (0.36**–**0.49)**	**0.54 (0.48**–**0.60)**
**Job status**				
No	1 (Reference)	1 (Reference)	1 (Reference)	1 (Reference)
Yes	**1.84 (1.25**–**2.69)**	**1.50 (1.09**–**2.06)**	**0.48 (0.34**–**0.67)**	**0.59 (0.46**–**0.75)**
**Marital status**				
Single	1 (Reference)		1 (Reference)	1 (Reference)
Living together/Married	**1.15 (0.86**–**1.53)**		**0.63 (0.51**–**0.76)**	**0.80 (0.71**–**0.90)**
Separate/Widow/Divorced	**1.39 (1.02**–**1.89)**		**0.62 (0.46**–**0.84)**	**0.75 (0.66**–**0.85)**

**Notes.**

* Multivariable models were created using backward elimination technique; variables for which there is no PR value in the adjusted model were dropped during the backward elimination process. Statistically significant results (*p* < 0.05) are in bold.

** The initial model included all the variables, sex, assets index and marital status were dropped because their *p*-value (Wald Test) was >0.05, thus the remaining variables were included in the multivariable model.

*** The initial model included all the variables and none were dropped because all were statistically significant for the Wald Test, thus all the variables were included in the multivariable model.

The indicators of socioeconomic disadvantage used in the analysis did not yield a unified direction in the relationships of interest. Different markers of socioeconomic status showed different directions of association with both outcomes, i.e., worse assets index and unemployment had opposite relationships with sleeping duration outcomes compared to the estimates obtained with lower education.

## Discussion

### Main findings

Few studies have assessed sleep duration at the population level in developing countries, and our study aimed to characterize the patterns of sleep duration in the Peruvian population taking advantage of a nationally representative cross-sectional survey. Our results indicate that the Peruvians’ self-reported sleep duration is similar to what is recommended by the National Sleep Foundation: sleep duration was, on average, close to 8 h, similar during weekdays and weekends. Relatively, urban habitants reported they slept less than their rural peers; this was constant either in weekdays or weekends. The socio-demographic profile characterizing short-sleepers differed from long-sleepers providing almost a mirror pattern between these two profiles, albeit with different magnitudes of association, in particular for factors such as age, education, assets and job status.

### Comparison with other studies

Average duration of sleep calculations were similar to those reported previously in international (Santos-Silva et al., 2010; Steptoe, Peacey & Wardle, 2006) and national (Calderón et al., 2010; Rey de Castro, Gallo & Loureiro, 2004; Rosales et al., 2009) studies. On average, sleep duration in other Latin American countries range from 7.2 h among Colombians (Steptoe, Peacey & Wardle, 2006), 7.3 in Venezuelans (Steptoe, Peacey & Wardle, 2006), and 7.5 in Brazilians (Santos-Silva et al., 2010). Previous Peruvian studies have reported sleep duration ranging from 6.8 to 7.5 h among bus drivers (Rey de Castro, Gallo & Loureiro, 2004; Rosales et al., 2009). Another study applied the Pittsburgh Sleep Quality Index in a small sample of people from the Andes, and reported mean sleep duration of 7 h (Calderón et al., 2010). Our observations expand the estimations available to-date to a population-based level.

When sleep duration was approached in short- and long-sleep categories, our results markedly differ from findings in other developed and developing countries. The prevalence of short-sleep during weekdays in the USA, despite using a lower cut-off (≤ 5 h), was 7.8% (Krueger & Friedman, 2009; Nunes et al., 2008). Studies from Finland (Kronholm et al., 2006) and Korea (Ryu, Kim & Han, 2011) report greater proportions of short-sleep, varying from 14.5% to 37.2%, respectively (both studies define short sleep duration as ≤ 6 h). Our study found a frequency of short-sleep duration of 4.3% during weekdays, much lower than the reported literature.

On the other hand, the prevalence of long-sleep in this study (22.4%) was much greater than equivalent estimates reported in the USA (8.5%) (Krueger & Friedman, 2009), Finland (13.5%) (Kronholm et al., 2006), and Korea (4.0%) (Ryu, Kim & Han, 2011). A seasonal effect has been posited to explain some of the differences observed between countries, e.g., longer sleep duration in autumn compared with summer as suggested by Bin, Marshall & Glozier (2011) and by a study with children (Hjorth et al., 2013). However, Peru is situated near the Equator and daylight variations during the year are not substantial. As such seasonality would not affect our calculations of sleeping categories, and therefore it does not explain the differences observed between our estimates and other studies.

The socio-demographic profile of short- and long-sleep characterized in our study is also different from those previously reported with regards to employment status and educational attainment. Krueger & Friedman (2009), also in a population-based study in the USA, found that those not working had increased odds of both short- and long-sleep, whereas in our study we observed such similar pattern for long-sleep only and the opposite for short-sleep. In the present study, those less educated were less likely to be short-sleepers; in contrast, studies in the USA (Krueger & Friedman, 2009) and Australia (Magee, Iverson & Caputi, 2009) reported the opposite, those with completed high-school or higher education had lower probability of being short-sleepers. These observations from contexts of rapid emerging countries, such as Peru, depict the complexities of addressing socioeconomic assessments (Howe et al., 2012), in relation to health outcomes that would otherwise remain unobserved in studies from more developed and established societies.

### Strengths and limitations

The study benefits from the population-based nature and the use of data from a large sample size. However, this study has limitations that must be pointed out. First, the analysis was based on data collected through self-reports and prone to recall bias, a frequent limitation in large surveys. Nevertheless, good correlation between subjective and objective measurements of sleep duration has been described both in adults (Lauderdale et al., 2008) and adolescents (Wolfson et al., 2003). Second, the methodology followed to calculate the daily sleep duration (total sleep duration in a given week divided by five) could have biased the results; nonetheless, the fact that the results are comparable to previous local reports may account for appropriate internal validity; on the other hand, there was not any specific data on napping habits. Third, the cross-sectional design can show only association instead of causality, a limitation shared by all surveys. Fourth, a great number of missing values might bias our results and reduce possibility of inferring them at the population level; additionally there were differences when comparing some variables (area, age, education and assets index) between participants with complete and missing data for the outcome of interest. Finally, the ENUT did not provide information about other important variables that have been reported to be associated with either short or long sleep such as smoking status, alcohol consumption, ethnicity, or physical activity (Krueger & Friedman, 2009; Magee, Iverson & Caputi, 2009; Ryu, Kim & Han, 2011; Stranges et al., 2008). Future research would benefit from an intensive exploration of the sleeping patterns reported and important health-related outcomes, including sleeping problems, e.g., obstructive sleep apnea. Also, given the rapid socio-demographic transitions occurring in many low- and middle-income countries, variations of sleep patterns over time at the national level and their relationship with health outcomes deserve further monitoring and scrutiny.

### Relevance for public health policy

Translating epidemiologic research into health policy could be tough; and this could be particularly difficult since providing a sleep duration policy could be seen as a restriction in anyone’s freedom to use their time. Peru is going through an epidemiological transition, and so are other developing countries. In this vein, there is a change in the population demographical distribution leading to a higher proportion of adults and elderly. Both scenarios have led to a higher prevalence of NCDs. Consequently, further efforts should be taken to address modifiable risk factors, including sleep duration and other sleep problems.

Describing the sociodemographic profile of the Peruvian population with higher probability of short- or long-sleep may be useful to inform and to develop potential interventions; for instance, to focus on urban habitants as they reported shorter sleep duration. Possible strategies might include the education of people about the benefits of adequate sleep duration and of good quality, which could raise their awareness about their sleep health. As people work more hours, ideally we could anticipate that such shifts should not occur in detriment of their sleep duration, as previously reported (Basner et al., 2007; Kronholm et al., 2006). These principles have been acknowledged in the USA through their National Prevention Strategy: American’s Plan for Better Health and Wellness (National Prevention Council, 2011). Sleep health has been included among the topics and objectives of Healthy People 2020, a set of 10-year objectives to improve USA citizens’ health (US Department of Health and Human Services, 2013), and this study sets a baseline scenario to consider corresponding prevention avenues for Peru and related contexts.

### Conclusions

The Peruvian population sleeps around 8 h during weekdays and weekends. There is a much higher frequency of long-sleep in contrast to short-sleep, though the majority was regular sleepers. The socio-demographic profile of short- and long-sleeping patterns is different, not only within our study but also when compared to other settings. The profile description provided by this study might be useful to develop strategies to protect and improve advantageous sleeping habits in people with short sleep duration—e.g., older people and those in the highest asset index—or long sleep duration— e.g., people with no formal education or just having completed high school. Furthermore, for any intervention to be successful it should address the most frequent issue in a given context. In our case, urban settings presented a higher frequency of short sleep duration comparing with rural settings, possibly because of the different economical activities and lifestyle patterns between these two settings.
